# Insights into the Therapeutic Potential of Glucocorticoid Receptor Modulators for Neurodegenerative Diseases

**DOI:** 10.3390/ijms21062137

**Published:** 2020-03-20

**Authors:** Alejandro F. De Nicola, Maria Meyer, Rachida Guennoun, Michael Schumacher, Hazel Hunt, Joseph Belanoff, E. Ronald de Kloet, Maria Claudia Gonzalez Deniselle

**Affiliations:** 1 Laboratory of Neuroendocrine Biochemistry, Instituto de Biologia y Medicina Experimental-CONICET, Obligado 2490, 1428 Buenos Aires, Argentina; mariameyer1981@gmail.com (M.M.); gonzalezdeniselle@gmail.com (M.C.G.D.); 2 Dept. of Human Biochemistry, Faculty of Medicine, University of Buenos Aires, Paraguay 2155, 1425 Buenos Aires, Argentina; 3 U1195 Inserm and University Paris-Sud and University Paris Saclay 80 rue du Général Leclerc, 94276 Kremlin-Bicêtre, France; rachida.guennoun@inserm.fr (R.G.); michael.schumacher@inserm.fr (M.S.); 4 CORCEPT Therapeutics, 149 Commonwealth Dr, Menlo Park, CA 94025, USA; hhunt@corcept.com (H.H.); jbelanoff@corcept.com (J.B.); 5 Division of Endocrinology, Dept. of Internal Medicine, Leiden University Medical Center, Albinusdreef 2, 2333 ZA Leiden, The Netherlands; erdekloet@gmail.com; 6 Dept. of Physiology, Faculty of Medicine, University of Buenos Aires, Paraguay 2155, 1425 Buenos Aires, Argentina

**Keywords:** Wobbler mouse, amyotrophic lateral sclerosis, glucocorticoid receptor antagonist, neurodegeneration, neuroinflammation

## Abstract

Glucocorticoids are crucial for stress-coping, resilience, and adaptation. However, if the stress hormones become dysregulated, the vulnerability to stress-related diseases is enhanced. In this brief review, we discuss the role of glucocorticoids in the pathogenesis of neurodegenerative disorders in both human and animal models, and focus in particular on amyotrophic lateral sclerosis (ALS). For this purpose, we used the Wobbler animal model, which mimics much of the pathology of ALS including a dysfunctional hypothalamic–pituitary–adrenal axis. We discuss recent studies that demonstrated that the pathological cascade characteristic for motoneuron degeneration of ALS is mimicked in the genetically selected Wobbler mouse and can be attenuated by treatment with the selective glucocorticoid receptor antagonist (GRA) CORT113176. In long-term treatment (3 weeks) GRA attenuated progression of the behavioral, inflammatory, excitatory, and cell-death-signaling pathways while increasing the survival signal of serine–threonine kinase (pAkt). The action mechanism of the GRA may be either by interfering with GR deactivation or by restoring the balance between pro- and anti-inflammatory signaling pathways driven by the complementary mineralocorticoid receptor (MR)- and GR-mediated actions of corticosterone. Accordingly, GR antagonism may have clinical relevance for the treatment of neurodegenerative diseases.

## 1. Introduction

The global rise of life expectancy is increasing the incidence of neurodegenerative diseases. From the endocrine point of view, an intriguing feature of most human neurodegenerative diseases is the presence of glucocorticoid overdrive, as revealed by hyperactivity of the hypothalamic–pituitary–adrenal (HPA) axis, hypercortisolemia, and flattening of the cortisol circadian rhythm. The glucocorticoid overdrive applies to both human and experimental models of neurodegeneration, and is the topic of this review.

Glucocorticoids’ effects under physiological and pathological conditions are mediated by binding to glucocorticoid receptors (GR) and mineralocorticoid receptors (MR). In the central nervous system, GR show a ubiquitous distribution in neurons and glial cells: astrocytes, microglia, and oligodendrocytes [1]. In certain regions of the CNS such as the hippocampus, glucocorticoids also bind to MR, which show a more restricted distribution and higher affinity to the naturally occurring glucocorticoids than the GR [2,3,4]. MR are highly expressed in the limbic-frontocortical brain region [5,6]. Activation of these MR during stress increases excitability, modulates selection of coping style, and promotes learning [7]. GR are abundantly expressed in the hippocampus, prefrontal cortex, paraventricular nucleus (PVN) of the hypothalamus, amygdala, and spinal cord [8]. Steroid-activated GRs exert negative feedback inhibition on the hippocampus, of corticotrophin-releasing hormone synthesis and release from the PVN, and of pituitary ACTH synthesis and release following stress. GR activation facilitates recovery from stress and enhances motivational arousal and memory storage. Thus, MR and GR modulate in complementary fashion various aspects of memory, learning, and behavioral adaptation [7,9]. 

Glucocorticoids have profound effects on intermediary metabolism by stimulating gluconeogenesis in the liver and lipolysis in fat cells. Glucocorticoids also hamper insulin action in the skeletal muscle, thereby preventing glucose uptake [10]. Collectively, these actions mediated by GR make energy substrates available to the brain for mitochondria. Glucocorticoids affect mitochondrial function via their action on membrane potential, oxidative phosphorylation, and calcium sequestration, thereby playing a key role in excitotoxicity during chronic adversity [10]. While glucocorticoids’ effects on mitochondrial function have been linked so far to GR, immune and inflammatory processes depend on cooperative MR- and GR-mediated actions. Ever since Selye’s pendulum hypothesis [11], it is known that mineralocorticoids are pro-inflammatory and pro-immune, while GR activation governs the well-known anti-inflammatory and immunosuppressive actions of glucocorticoids. Glucocorticoids have profound effects on cardiovascular functions and fluid homeostasis, involving both MR- and GR-mediated actions and depending on the presence of the 11β-hydroxysteroid dehydrogenases (HSD) (Box 1).

Box 1 **11β-hydroxysteroid dehydrogenase type 1 and 2 (HSD1 and 2).**

HSD1 is a NADPH-dependent reductase which regenerates bioactive glucocorticoid cortisol and corticosterone from the inactive 11-keto congeners. HSD1 is expressed in the brain and heart. HSD2 is a NAD+-dependent oxidase that inactivates cortisol and corticosterone by converting these steroids into 11 inactive ketosteroids: cortisone and 11-dehydrocorticosterone. HSD2 is expressed in vascular endothelial cells, epithelial cells of the kidney, intestines, and salivary glands. In the brain, expression is limited to n. tractus solitarii (NTS) and periventricular areas. The glucocorticoids cortisol and corticosterone circulate in a 100- to 1000-fold excess over aldosterone. The inactivation of bioactive glucocorticoids by HSD2 renders the MR aldosterone-selective in control of salt homeostasis from salt appetite by the NTS neuronal network to salt retention by the kidney [12].

Glucocorticoid actions in the spinal cord come to light mainly after injury, including effects on myelination, oligodendrocyte protection, anti-nociception, anti-inflammatory effects, and control of growth factors and enzymes essential for motoneuron function [13,14]. Conversely, chronic glucocorticoid exposure in a particular pro-inflammatory or degenerative environment becomes damaging, as is further discussed in this review. 

As already mentioned, several neurodegenerative diseases involve glucocorticoid overdrive. In Alzheimer’s disease, a chronic form of dementia caused by extracellular deposits of β-amyloid and intracellular tau hyperphosphorylation, there are increased levels of cortisol in the cerebrospinal fluid and dysregulation of the HPA axis [15,16,17]. In Parkinson’s disease, the progressive loss of dopaminergic neurons of the substantia nigra is accompanied by higher diurnal cortisol levels and changes of the cortisol circadian rhythm [18]. Hyperactivity of the HPA axis has been reported in Huntington’s patients [19], who suffer from a triplet repeat expansion of the huntingtin protein gene. Multiple sclerosis (MS) is the most common demyelinating disease, in which neuroinflammation and neurodegeneration play major roles in illness progression [20]. MS patients show abnormal cortisol secretion, HPA axis hyperactivity, and a negative dexamethasone suppression test [21]. Endocrine dysfunction has also been reported in amyotrophic lateral sclerosis (ALS), a fatal, progressive neurodegenerative disease that affects the motoneurons of the brain and spinal cord. ALS patients show elevated cortisol in plasma and saliva, loss of circadian rhythm, hypo-responsiveness to mild stress, and a blunted cortisol awakening responses [22,23,24,25]. 

There are additional examples of glucocorticoid overdrive and changes of CNS function or chemistry. One of these is metabolic syndrome (a tetrad of hypertriglyceridemia, hypertension, obesity, and hyperglycemia). Patients with metabolic syndrome show poor cognition, oxidative stress, and neuroinflammation, accompanied by hypercortisolemia, a highly reactive HPA axis, and dysregulation of the cortisol-producing enzyme HSD1 [26]. There are mutations of the GR, altering its function in metabolic syndrome [27]. 

Depression is also comorbid with neurodegeneration [28], and patients with depression, in particular if their depression is accompanied by psychotic symptoms, show resistance to glucocorticoid suppression of the HPA axis and high levels of circulating cortisol and ACTH [27,28]. Another example comes from patients with stroke, a circumstance leading to secondary neurodegeneration. Stroke causes hypercortisolemia plus decline in cognition, low levels of neurotrophic factors, and increased markers of oxidative stress [29,30]. Thus, the concomitant changes in the production, action, central regulation, and rhythm of glucocorticoids with changes of brain pathology have led to cortisol being proposed as a biomarker of human neurodegeneration [15].

## 2. Hypercorticosteronemia in Experimental Neurodegenerative Disorders 

Similarly to results from human studies, substantial dysregulation of adrenal function has been reported in animal models of neurodegenerative diseases. In familial Alzheimer’s disease modeled by transgenic mice, there is an abnormal glucocorticoid cycle (i.e., HPA axis hyperactivity, high corticosterone levels) which may enhance β-amyloid deposition and release [31]. In early symptomatic Tg2576 mice, Lante et al. [32] reported compromised HPA axis, altered corticosterone circadian rhythm, and feedback mechanisms concomitant with brain changes resembling human Alzheimer’s pathology. A cross-talk between GR and β-amyloid has been found in the hippocampus, because this peptide increases the GR in spines and post-synaptic densities and impairs long-term potentiation [33,34]. Furthermore, there is a GR-responsive element (GRE) on the promoter of the amyloid precursor protein (APP) and in beta-site APP cleaving enzyme [35], suggesting direct glucocorticoid effects at the gene level. 

In the R6/2 transgenic mice model of Huntington’s neurodegeneration, there is adrenal hypertrophy, increases in ACTH and corticosterone in plasma coupled to muscular atrophy, insulin resistance, and fat deposition, suggesting a Cushing’s-like condition [36]. Moreover, at the early stages of an experimental autoimmune encephalomyelitis (EAE) model of MS, immunized mice showed increased plasma corticosterone, changes of the HPA axis, brain neuroinflammation, and emotional and cognitive decline [37]. Additionally, in a model of Parkinson’s disease produced by injection of an adenovirus vector expressing α-synuclein into the substantia nigra, rats developed a hyperactive HPA axis regulation of corticosterone [38]. The authors [38] provided evidence that in experimental Parkinsonism, depression is closely related to dysregulation of the HPA axis. 

Models of ALS also provide substantial evidence for the role of glucocorticoids in neurodegeneration. In the Wobbler mouse, motoneuron degeneration due to a point mutation of Vps54 (vesicular vacuolar protein sorting 54) impairs the retrograde vesicle transport and Golgi function [39]. Wobblers show adrenal hypertrophy, high plasma and tissue corticosterone levels [40,41], neuroinflammation, micro- and astrogliosis, increased excitatory neurotransmission, and changes of glutamate synthesis and glutamate transporters in the brain and spinal cord [42,43,44,45]. The dysfunctional adrenal steroid system of the Wobbler is shared by the superoxide dismutase (SOD1) transgenic mouse model of familial ALS. SOD1 transgenics present aberrant serum corticosterone circadian rhythm and high circulating steroid levels [46]. In another model of ALS, the TDP43 (transactive response DNA-binding protein) mouse, glucocorticoids enhance neurotoxicity [47].

Finally, transgenic mice overexpressing HSD type 1 (HSD1, see Box 1), a corticosterone-regenerating enzyme, show clinical features of metabolic syndrome (obesity, insulin resistance, dyslipidemia) suggesting a role for glucocorticoids in the pathogenesis of metabolic syndrome [48]. It has been shown that in metabolic syndrome, a NR3C1 polymorphism of the GR predisposes to HPA dysregulation [49]. Metabolic syndrome also impairs vascular reactivity and causes neuroinflammation and oxidative stress in the CNS, providing a substrate for neurodegeneration [26]. In conclusion, data obtained in animal models of human diseases support the hypothesis that increased corticosterone may be a signature of neurodegeneration [41]. 

## 3. Glucocorticoids, Inflammation, and CNS Disorders

Glucocorticoids exert many effects in the periphery and the CNS. Traditionally, these steroids have been used in the clinical practice due to their potent anti-inflammatory and immunosuppressive actions. These properties makes glucocorticoids pharmacological tools for the treatment of asthma, rheumatoid arthritis, skin diseases, psoriasis and eczema, hematological cancers, inflammatory diseases of the bowel, autoimmune MS, and secondary effects of CNS trauma, and for prevention of transplant rejection [50,51] 

However, glucocorticoids not always exert anti-inflammatory and immunosuppressive effects. For example, opposite and even detrimental effects were observed in the CNS when steroid levels were elevated after stress or after chronic administration in a prevailing inflammatory environment or in certain mutations of the GR. Thus, chronic treatment of rodents with high doses of corticosterone increased reactive oxygen species, as reviewed in Reference [52], and increased cytokines and pro-inflammatory factors in the hippocampus in the kainic acid model of excitotoxicity [53]. These reports suggest an enhanced, not suppressive, response of the central immune system. In this context, prolonged glucocorticoid elevation becomes a danger signal or alarmin, priming microglia to respond to LPS by increasing TNFα, interleukins, and the inflammasome NLPR3 (NLR Family Pyrin Domain Containing 3) [54]. Glucocorticoids are also detrimental in cuprizone-induced demyelination, in which they activate microglia to produce pro-inflammatory mediators [55]. The synthetic agonist dexamethasone increases activation and proliferation of microglia in the CA1 and CA3 regions of hippocampus of 3XTg Alzheimer’s mice, resulting in decreased density of spines and behavioral changes [56]. 

After peripheral nerve injury, glucocorticoids increase allodynia and the n-methyl-D-aspartate (NMDA) receptor, effects associated with inflammation [57]. In the hippocampus, excess levels of glucocorticoids activate the NLPR1 inflammasome [54] and induce hyperactivity of glutamatergic neurotransmission [53]. Thus, a heightened inflammatory response of the CNS and PNS accompanies glucocorticoid overexposure. In this context, glucocorticoids have been considered a double-edged sword because they mediate both anti-inflammatory and pro-inflammatory effects [50,51]. 

In conclusion, neurodegeneration, neuroinflammation, stress, and excitoxicity constitute a pathological background in which glucocorticoids promote a pro-inflammatory state [58]. Such a pro-inflammatory state could be caused by an imbalance in the pro- vs. anti-inflammatory action of the natural glucocorticoids, as these are mediated by MR and GR. Previous saturation analysis has shown a non-significant reduction in Bmax with increased Kd for the GR in the spinal cord of Wobbler mice. However, in the hippocampus, there is a downregulation of GR, a probable response to increased corticosterone levels [40]. Thus, overexposure to glucocorticoids may cause a downregulation of GR, leading to a relative over-activity of the MR. If so, holding back the glucocorticoid overdrive, causing downregulation of the GR, might be therapeutically useful to reduce neuropathology. 

## 4. Effect of Glucocorticoid Receptor Antagonists in CNS Disorders 

The previously mentioned literature references suggest that GR antagonists (GRA) should prevent inflammatory effects caused by glucocorticoid overdrive. Mifepristone, also known as RU486 (11-[4-(dimethylamino)phenyl]-17-hydroxy-17-[1-propynyl]-[11β,17β]-oestra-4,9-diene-3-one), has been the conventional treatment for human and experimental diseases involving the GR. Mifepristone at higher doses than needed for progesterone antagonism shows an 18-fold higher affinity for GR than cortisol [59]. However, in addition to exerting anti-glucocorticoid activity, mifepristone also binds to progesterone receptors and weakly to androgen receptors [59]. In spite of pitfalls in specificity, mifepristone has been an effective drug for the treatment of endogenous Cushing’s syndrome of all etiologies (Box 2).

Box 2 **Mifepristone (RU 486).**

Mifepristone has been shown to be clinically effective in the treatment of pathological conditions characterized by hypercortisolemia [60]. In the search for its underlying mechanism of action, rodent models showed surprising results, which were extensively discussed in a recent report [61]. In rodents, mifepristone is readily degraded and poorly penetrates the brain. Hence, a 100,000-fold higher mifepristone dose is needed systemically as compared to intra-cerebro-ventricularly (icv) in order to evoke a behavioral effect. The endocrinology of mifepristone is remarkable; after one administration, the expected disinhibition of the HPA axis occurs with at least a 12 h period of corticosterone hypersecretion, and up to 24 h the mice are hyper-responsive to a stressor. However, this expected hypercorticosteronemia disappears entirely after repeated daily mifepristone administration. After 7 days of mifepristone administration, circulating corticosterone remains comparable with controls, and the stress response is half that of the control animals and 7-fold lower than the group administered mifepristone for 1 day. The cause of this rapid reset of the endocrine and behavioral stress response may be (i) a long-term rebound glucocorticoid feedback after mifepristone, (ii) a partial agonist effect of mifepristone upon recruitment of the GR coregulator cocktail, or (iii) a more prominent tonic-MR-mediated control of limbic circuits inhibitory to the stress response. Interestingly, this mifepristone-induced reset of the stress system is reminiscent of conditions wherein the mandatory glucocorticoid increase after a stressor is prevented by, for example, rapid removal of the adrenals. For weeks, stress sensitivity is dramatically increased, unless glucocorticoids are given post-stress to mimic the glucocorticoid stress response [62].

Mifepristone has also been considered as an adjunctive therapy to slow or reverse the progression of cognitive decline in Alzheimer’s disease, considering the hyperactivity of the HPA axis and high cortisol levels of these patients [63]. Antagonism of the GR with mifepristone also produces beneficial effects in animal models, as revealed by the decreased vulnerability of hippocampal neurons subjected to β-amyloid burden [64]. In 3xTg Alzheimer mice, mifepristone reduced β-amyloid and tau pathology [65], restored spine density, and enhanced behavioral performance of dexamethasone-treated 3xTg Alzheimer mice [50], blocked the effect of stress on the inflammatory molecule HMGB1 [54], normalized the stress-induced reduction of neurogenesis [66], attenuated inflammation and microglia activation caused by intracerebroventricular injection of IL1β [67], and mitigated hippocampus pathology of status epilepticus [68]. The effects of mifepristone has also been observed in models of type I diabetes, in which excessive GR overstimulation induces hippocampus pathology. Thus, treatment of streptozotocin-diabetic rats with mifepristone decreases hippocampus pathology and prevents cognitive impairment [69]. These reports indicate the usefulness of mifepristone to inhibit the glucocorticoid overdrive shown in different CNS pathologies.

More recently, new anti-glucocorticoids with higher specificity and affinity for the GR have been synthesized and their efficacy studied in different animal models of CNS disorders. Thus, CORT108297 (4Ar)-4a-(ethoxymethyl)-1-(4-fluorophenyl)-6-[4-(trifluoromethyl)phenyl]sulfonyl-4,5,7,8-tetrahydropyrazolo [3–g]isoquinoline) prevents the glucocorticoid-induced decrease of hippocampus neurogenesis in normal mice, normal rats, and Wobbler mice [43,70]. CORT108297 also inhibits diet-induced obesity and inflammation [71] and, according to Solomon et al. [72], may be an alternative therapy to attenuate the stress responses and behavioral changes associated with forced swim stress in rodents. In Alzheimer’s transgenic mice, detrimental and toxic effects due to β-amyloid_25–35_ peptide accumulation disappeared after treatment with the GR antagonists CORT108297 and CORT113176 [73]. The therapeutic potential of CORT113176 (Box 3) has been studied in the Wobbler mouse. Given for short or prolonged periods substantially reduced the motoneuron degeneration and gliosis (astro and microgliosis) of Wobbler mice [43,74].

Box 3 **Structure and properties of CORT113176.**

The chemical structure of CORT113176 is ([4a(R)-1-(4-fluorophenyl)-6-(4-trifluoromethylphenyl)sulfonyl)-4,4a,5,6,7,8-hexahydro-4aH-pyrazolo[3,4-g]isoquinolin-4a-yl][pyridine 2yl]methanone1H-pyrazolo). This specific modulator of the glucocorticoid receptor (GR) shows a Ki value of 0.26 nM for the GR in vitro, but presents low or negligible binding efficiencies for the MR, progesterone receptor, androgen receptor and estrogen receptor in human HepG2 and rat H4 cells [75]. In Alzheimer’s mice CORT113176 prevented accumulation and toxic effects of β-amyloid [73], whereas in the Wobbler mouse it reduced motoneuron degeneration and showed anti-inflammatory effects [43,74]. Ouside the CNS, CORT113176 decreases glucocorticoid unwanted metabolic effects in a model of type II diabetes [75].

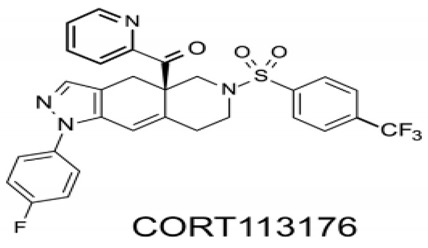

Another synthetic compound, CORT118335 6-(4-phenylcyclohexyl)-5-[[3-(trifluoromethyl)phenyl]methyl]-1*H*-pyrimidine-2,4-dione), shows high affinity for GR, and moderate affinity for MR without binding to progesterone or androgen receptors. CORT118335 is a MR/GR antagonist in the hippocampus, where it antagonizes the mRNA of several glucocorticoid-induced genes. It shows GR agonist activity on the structural components of the HPA axis [75]. Thus, CORT118335 decreases ACTH and corticosterone responses to forced swim stress and up-regulates c-Fos levels in the hypothalamus, supporting its value as an antidepressant agent [74]. CORT118135 also has effects outside the CNS including in the adipocytes, in which it reduces weight gain and fat expansion due to a high-fat diet [76].

## 5. Efficacy of a Glucocorticoid Receptor Antagonist in Wobbler Neurodegeneration

We studied the effects of a GRA in the Wobbler mouser model of sporadic ALS. These mutants show endocrine dysfunction, and in particular high levels of corticosterone. These levels were 3.5× in plasma, 2× in brain, 4.5× in spinal cord (cervical, thoracic, and lumbar regions), and 5× higher in adrenal glands than in control mice, measured by a sensitive and specific GC/MS method [41]. The high levels of corticosterone were accompanied by signs of zonal hypertrophy of the adrenal glands—in particular, increase of the ratio of adrenal gland/body weight and the size of some cells of the zona fasciculata, the main site of corticosterone synthesis. The high levels of corticosterone in brain and spinal cord may contribute to the motoneuron degeneration and hippocampus vulnerability in Wobbler mice. The development of motoneuron vacuolation, accompanied by spinal cord gliosis and neuroinflammation, make Wobblers an ideal model with which to analyze the effects of GRA in a combined neurodegeneration/neuroinflammation/hypercorticosteronemia model.

**Figure 1:**
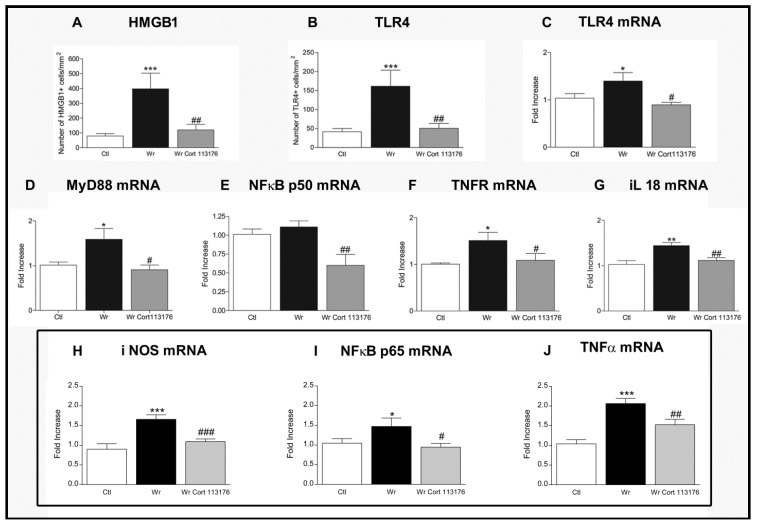
(**A**–**G**), Effects of 3 weeks treatment with CORT113176 on inflammatory factors in Wobbler mice (Wr) spinal cord. Control mice (CTL) are represented by white columns, vehicle-injected Wr by dark columns, and CORT113176-treated Wr mice by gray columns. Wr mice expressed higher numbers of the high mobility group box 1 protein (HMGB1)+ cells (**A**) and toll-like receptor 4 (TLR4) + cells (**B**). Wr mice also showed higher levels of mRNA for TLR_4_ (**C**), myeloid differentiation primary response 88 (MyD88) (**D**), and slightly higher levels for p50 subunit of nuclear factor kappa B (NFκB) (**E**). mRNAs of tumor necrosis factor receptor 1 (TNFR1) (**F**) and IL(interleukin) -18 (**G**) (* *p* < 0.05; ** *p* < 0.01; *** *p* < 0.001 vs. CTL mice). CORT113176 treatment of Wr mice for 3 weeks decreased the number of HMGB1+ cells and TLR_4_+ cells (**A**,**B**), and the five mRNAs for TLR4, MyD88, NFκB, TNFR, and IL-18 (**C**–**G**) (^#^ *p* < 0.05; ^##^ *p* < 0.01 vs. untreated Wr). The three graphs inside the rectangle represent data from CTL, untreated Wr, and Wr receiving a 4 day treatment with CORT113176. Wr mice showed higher levels of mRNA for inducible nitric oxide synthase (iNOS) (**H**), p65 subunit of NFκB (**I**), and tumor necrosis factor α (TNFα) (**J**) (* *p* < 0.05; *** *p* < 0.001 vs. CTL mice). CORT113176 reduced iNOS, NFκB, and TNFα mRNAs (^#^ *p* < 0.05; ^##^ *p* < 0.01; ^###^ *p* < 0.01 vs untreated Wr). Results are plotted as relative changes (mean ±SEM) with control mRNAs taken as 1.0. Figure modified from Reference [77].

Increased number of glial cells of the astrocyte and microglia lineage, plus increased expression of pro-inflamamtory markers, confirmed a high degree inflammation in Wobbler mouse spinal cord [44,77] (Figure 1). Accordingly, Wobblers showed increased number of astrocytes labeled for glial fibrillary acidic protein (GFAP), and increased expression of the microglia markers Iba1 and cluster of differentiation molecule 11 (CD11B) mRNA. These changes in the density and functional phenotype of microglia and astrocytes suggest that they are the source of toxic inflammatory mediators striking the ailing motoneurons [78]. The analysis of mRNA expression by real-time polymerase chain reaction techniques showed changes of the inflammatory mediator HMGB1, a danger-asssociated molecular pattern molecule overexpressed by Wobbler mice in common with SOD1 transgenic mice and ALS patients [79,80]. Neurotoxicity of HMGB1 requieres binding to the toll-like receptor 4 (TLR4) [81], which is also upregulated in Wobbler spinal cord. Other pro-inflammatory molecules are concurrently increased in Wobbler spinal cord, such as the myeloid differentiation primary response (MyD88) and the p50/p65 subunits of NFκB [44,77]. NFκB becomes the final downstream signaling molecules of the HMGB1/TLR4 pathway. Nuclear genomic effects of NFκB then prime the NLRP3 inflammasome and increase the transcription of genes dependent on this transcription factor, such as NLRP3 and the inactive precursors of interleukins pro-IL1β and pro-IL18 [82]. In turn, caspase 1 activation by the inflammasome cleaves the precursor proteins into mature IL-1β and IL-18, which are secreted by inflammatory cells [83]. IL18 represents a molecule of interest because it is elevated in untreated Wobbler mice, and also stimulates the synthesis and release of HMGB1 by microglia of ALS patients [83].

Therefore, it is likely that GR activation by the chronically elevated corticosterone of Wobbler mice modulates glial cells to increase HMGB1, TLR4, MyD88, NFκB, and IL18, constituting a positive feedback loop mantaining chronic inflammation of the spinal cord (Figure 1). This cycle shows a high degree of plasticity, and can be interrupted by blockage of the GR. To this end, CORT113176 was given to male and female Wobbler mice at the dose of 30 mg/kg for 3 weeks, at the end of which we analyzed whether the glial-derived pro-inflammatory mediators were curtailed. After this dose and period of treatment, we found that CORT113176 decreased astro and microgliosis, and downregulated the inflammatory mediator HMGB1 and its cognate receptor TLR4 in immunoreactive cells showing a glial morphology. The GRA also decreased the expression of TLR4, MyD88 mRNA, TNFR, and the p50 subunit of NFκB, and, at the end of the mentioned loop, downregulated IL18 mRNA [75] (Figure 1).

**Figure 2:**
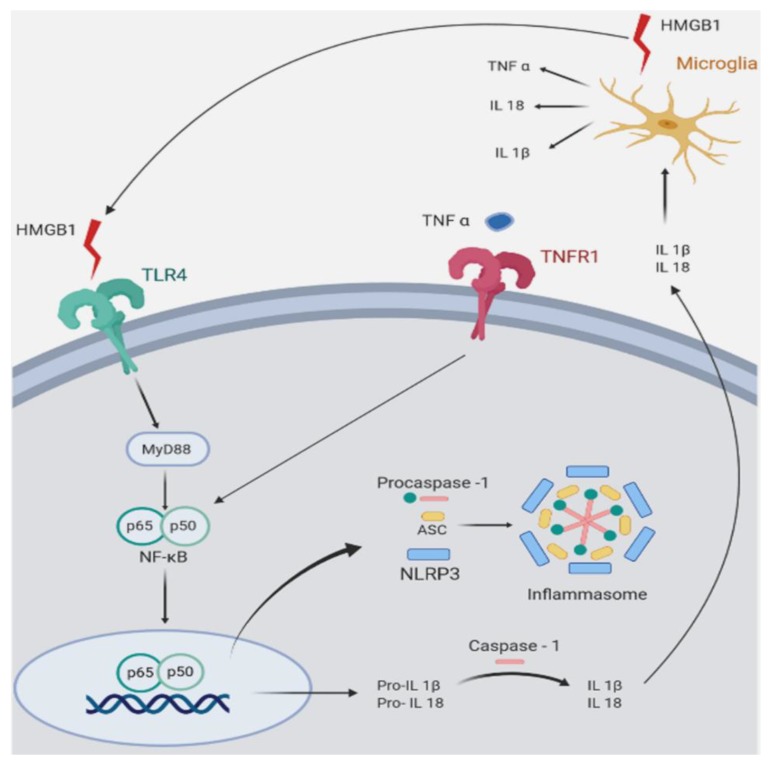
Symplified representation of pro-inflammatory mediators in Wobbler (Wr) mouse spinal cord. The figure shows inflammatory pathways that are upregulated in Wr mice. Wr mice show increased expression of the high mobility group box 1 alarmin (HMGB1), a ligand of the toll-like receptor 4 (TLR4). The signaling pathway of TLR4 involves MyD88 (myeloid differentiation primary response 88), an adapter protein that activates nuclear translocation of the p65/p50 subunits of NFκB (nuclear factor kappa B). In Wr mice, the NFκB pathway activates and assembles the inflammasome (an intracellular multi-protein complex) and stimulates transcription of the inflammasome component NLRP3 (NLR family pyrin domain containing 3), and of the pro-interleukins 1β and 18. Both are converted by caspase 1 into mature IL-1β and IL18, which are secreted and stimulate microglial cells to further produce HMGB1, TNFα, IL-18, and IL-1β. The similar effect on caspase 1 is produced by TNFα binding to its cognate receptor TNFR1. The glucocorticoid receptor antagonist (GRA) CORT113176 shows inhibitory effects on HMGB1, TLR4, MyD88, p50/p65 subunits of NFκB, TNFα, TNFR1, and IL18. Quantitative aspects of CORT113176 effects are shown in Figure 1. CORT113176 also reduces the density of microglia and astrocytes, cells of origin of the inflammatory mediators. In consequence, CORT113176 inhibits pro-inflammatory factors and their cells of origin in the Wr spinal cord. (Figure modified from Liu et al. [83]; data from Wr mice ± CORT113176 modified from Meyer et al. [44,75].)

In summary, reduction of pro-inflammatory markers was successfully achieved after prolonged treatment with CORT113176. We also reported that pro-inflammatory markers in Wobbler spinal cord responded similarly after short-term treatment with CORT113176 [44]. In particular, 4 days of treatment with CORT113176 decreased the expression of the inducible nitric oxide synthase (iNOS), p65 subunit of NFκB and TNFα mRNAs, among other factors [44] (Figure 1). Figure 2 summarizes the hypothetical targets of CORT113176 along the mentioned inflammatory pathway in the Wobbler spinal cord.

CORT113176’s effects are not limited to restrictive effects on pro-inflammatory markers of the Wobbler spinal cord. Significant changes are also observed in the cell death/survival pathways AKT and JNK family of kinases, which are involved in neurodegeneration [84]. A reduced pAKT/AKT ratio and increased pJNK/JNK ratio appears in the spinal cord of vehicle-treated Wobblers, suggesting apoptosis. Interestingly, treatment of Wobbler mice with CORT113176 produces the opposite effect, with increases in pAKT/AKT and decreases of the pJNK/JNK ratio. Thus, CORT113176 supports pro-survival and opposes anti-apoptotic pathways [77]. A third set of data emerged from the measurement of several elements of the glutamatergic system. In this regard, Wobbler mice showed abnormalities of glutamate homeostasis, resulting in glutamate excitotoxicity which also triggered motoneuron damage [42,45]. CORT113176 treatment restored glutamate homeostasis because it increases the glutamate-inactivating enzyme glutamine synthase and increased glutamate transporters [75]. These transporters, labeled GLT1 and GLAST in the mouse, deprived the synapses of excess glutamate, thus decreasing glutamate excitotoxicity.

In addition to neurochemical improvements, we also studied whether CORT113176 produces changes of motor behavior and paw anatomy in the Wobbler mouse. A first attempt employing a short 4 day course of CORT113176 treatment failed to change these parameters, although inflammatory markers and glial reactivity were reduced and motoneuron degeneration was attenuated [44]. In a second study, we found enhanced resistance to fatigue, increased motor performance, and lower forelimb atrophy when CORT113176 therapy was applied for 3 weeks [77]. These preclinical data highlight the possible translational value of prolonged treatment with CORT113176. However, it should be considered that there are sex differences in stress-related psychiatric disorders and neurodegenerative diseases, including ALS. Thus, anxiety and depression and some addictive behaviors are female-prevalent, while prevalence in males is higher for schizophrenia, autism, attention deficit hyperactivity disorder, and drug-related addictive behaviors [85]. There are also profound sex differences in the HPA axis response to stress in these disorders. During depression and anxiety disorders, stress-induced cortisol responses are elevated in males, but suppressed in females as compared to healthy controls [86]. These differences are related to the sex steroids. Estrogens are known to suppress stress-induced HPA axis activity, while this is increased by androgens and these sex differences are reflected in glucocorticoid feedback efficacy. Indeed, in the liver, glucocorticoid-responsive genes are overrepresented in males as compared to females, and this difference seems to extend to the brain [87]. The lifetime risk for ALS is higher for males than for females, particularly because men predominate in younger age groups [2,84]. Therefore, the possibility for sex differences in the response to GRA should be further studied.

## 6. GRA and Neurodegenerative Diseases: Lessons from Animal Models

There is now growing evidence that patients with neurodegenerative diseases and models of neurodegeneration show widespread disturbances of the HPA axis that may accelerate initiation and/or progression of the distinctive pathology. An open dilemma is to unveil the mechanisms of neurodegenerative diseases that stimulate the HPA axis, increase plasma glucocorticoids, flatten circadian rhythms, and induce resistance to the negative feedback of glucocorticoids. On one hand, glucocorticoids hinder the stimulatory response of peripheral cytokines after an immune challenge, but on the other hand, the same cytokines stimulate adrenal glucocorticoid production, acting at several CNS levels. Thus, IL-1β, IL6, TNFα, and other inflammatory mediators (nitric oxide and products of cyclooxygenase 2, etc.) modulate the function of the HPA axis at different levels of the CNS and/or pituitary gland [84].

The final components, the glucocorticoids, normally oppose the expression of immune mediators increasing during peripheral or central inflammation. In neurodegenerative diseases, the comorbid association with neuroinflammation activates glial cells to increase the local production of pro-inflammatory factors. These factors induce a pathological phenotype in microglia and astrocytes, leading to neuronal and oligodendrocyte damage. In this regard, receptors for cytokines and pro-inflammatory factors are present in several regions of the CNS, including those pertaining to the HPA axis [84]. Therefore, pro-inflammatory mediators produced in neurodegenerative diseases trigger HPA activation, increasing hypothalamic corticotrophin-releasing factor, vasopressin, and pituitary ACTH [84]. Thus, inflammatory mediators activate the HPA axis and facilitate chronic glucocorticoid pro-inflammatory effects in a permissive neurodegenerative environment.

In order to hold back this vicious circle, results obtained with the GRAs draw attention to the GR as a therapeutic target for neurodegenerative and neuroinflammatory diseases. In this review, we focused on the efficacy of several GRAs to decrease the neuropathology and behavior of Alzheimer’s transgenic mice and obesity-induced neuroinflammation, to block the reduction of neurogenesis in corticosterone-treated mice and rats, to modulate the activity of the HPA axis, and to attenuate stress-induced depression. Most of our own studies have been concentrated on the effects of CORT113176 in the Wobbler mouse model of ALS, because this mouse model shows ongoing inflammation coexisting with hypercorticosteronemia. In Wobbler mice, treatment with CORT113176 produces anti-inflammatory effects and anti-apoptotic actions, restores glutamatergic homeostasis, and decreases motoneuron degeneration in the spinal cord. In spite of the fact that present data is mostly experimental, it is hoped that clinical trials employing GRA may soon be available to test the beneficial effects of novel GRA in humans with neurodegenerative diseases.

## 7. Concluding Remarks

Neurodegenerative diseases are characterized by excess circulating cortisol, and ALS is no exception. ALS patients show a flattened elevated daily cortisol rhythm as a hallmark of hypercortisolemia [22,23,24,25]. We demonstrated in an animal model for ALS (the Wobbler mouse) that signs of spinal motoneuron degeneration and its accompanying behavioral phenotype can be halted by a 3 week course with the selective GRA CORT113176. This finding shows, at least in animals, that deactivation of the GR and/or recovery of the MR:GR balance may be a neuropharmaceutical treatment strategy for attenuating the damaging impact of hypercortisolemia in neurodegenerative diseases. Outstanding questions include precisely how this beneficial effect of the GRA in this animal occurs and, importantly, whether these promising results can be extended to ALS patients.

## Abbreviations

ACTH	adrenocorticotrophic hormone
AKT	a serine/thronine kinase
ALS	amyotrophic lateral sclerosis
APP	amyloid precursor protein
CD11b	cluster of differentiation molecule 11 B
CNS	central nervous system
EAE	experimental autoimmune encephalomyelitis
GC/MS	gas chromatography /mass spectrometry
GFAP	glial fibrillary acidic protein
GLAST	glutamate/aspartate transporter
GLT1	glutamate transporter 1
GR	glucocorticoid receptor
GRA	glucocorticoid receptor antagonist
GRE	glucocorticoid response element
HMGB1	high mobility group box 1 protein
HPA	hypothalamic pituitary adrenal axis
HSD	11β-hydroxusteroid dehydrogenase
IL18	interleukin 18
IL1β	interleukin 1 beta
IL6	interleukin 6
JNK	c-Jun-N-terminal kinase
MR	mineralocorticoid receptor
MS	multiple sclerosis
MyD88	myeloid differentiation primary response 88
NFκB	nuclear factor kappa B
NLPR3	NLR family pyrin domain containing 3 inflammasome
NMDA	n-metyl-D-aspartate
iNOS	nitric oxide synthase
PVN	paraventricular nucleus
SOD1	superoxide dismutase 1
TDP43	transactive response DNA-binding protein 42
TLR4	toll-like receptor 4
TNFR	tumor necrosis factor receptor
TNFα	tumor necrosis factor alpha
Vps54	vesicular vacuolar protein sorting 54
